# Personalized models of eating disorder symptoms and suicide risk in anorexia nervosa spectrum disorders: protocol for a longitudinal, passive-sensing study

**DOI:** 10.1186/s40359-025-03627-7

**Published:** 2025-11-26

**Authors:** Lauren M. Harris, Rachel Torres, Elizabeth D. Cash, April R. Smith, Cheri A. Levinson

**Affiliations:** 1 Department of Psychological Sciences, Auburn University, Auburn, AL USA; 2 Department of Psychological and Brain Sciences, University of Louisville, Louisville, KY USA; 3 Department of Otolaryngology, Head and Neck Survey & Communication Disorders, University of Louisville School of Medicine, Louisville, KY USA; 4 University of Louisville Health, Brown Cancer Center, Louisville, KY USA; 5 Department of Pediatrics, Division of Child and Adolescent Psychiatry and Psychology, University of Louisville School of Medicine, Louisville, KY USA

**Keywords:** Anorexia nervosa, Atypical anorexia, Suicide, Ecological momentary assessment, Passive sensing

## Abstract

**Background:**

Individuals with Anorexia Nervosa and Atypical Anorexia (termed here Anorexia Nervosa Spectrum Disorder, or ANSD) are at elevated suicide risk, yet limited research has explored the progression from suicidal ideation to suicide attempts among individuals with ANSD. Leveraging intensive real-time data, the present study will identify the behavioral, physiological, and psychological mechanisms linking ANSD and suicide.

**Methods:**

Our sample will comprise 230 adults with ANSD and current and/or lifetime suicidality. Participants will complete 21 days of ecological momentary assessment while wearing a sensor band that continuously monitors physiological indices of emotion regulation and arousal. Follow-up assessments will be conducted at one month, six months, and 12 months after baseline. Using a novel network analytic approach, we will integrate self-report and physiological data to develop group- and individual-level symptom models. These models will allow us to identify features that (1) maintain comorbid ANSD and suicidality, and (2) predict future suicidal thoughts and behaviors, at both the group and individual level.

**Discussion:**

Ultimately, findings from this project will facilitate the development of highly personalized intervention strategies that prevent suicidal behaviors before they occur, as well as personalized treatments that effectively treat ANSD symptoms in real-time.

## Suicide across the Anorexia Nervosa spectrum

Anorexia Nervosa (AN) is a serious eating disorder [1] characterized by adverse physical health effects [2] and high mortality [3]. Although the elevated mortality rates observed in AN are partially a consequence of medical complications secondary to malnutrition, suicide is responsible for approximately 20% of deaths observed in AN [3]. Atypical Anorexia Nervosa (AAN), a disorder characterized by the same symptoms as AN with the exception of underweight status [1], is indistinguishable from AN on the basis of suicidal ideation and suicide attempt history [4]. AAN also appears to be more common than AN; whereas AN affects approximately 4% of women and 0.3% of men [5], estimated prevalence rates of AAN approach 13% [6], underscoring the importance of identifying risk factors for suicide in this population.

The common co-occurrence of AN/AAN (termed here Anorexia Nervosa Spectrum Disorder, or ANSD) and suicidality may reflect shared etiological or maintenance processes. For example, restrictive eating is central to the development and persistence of ANSD [7] and is also a significant predictor of suicidal ideation among those with low-weight eating disorders, over and above the risk conferred by binge eating and purging [8]. Similarly, body dissatisfaction, which encompasses a range of negative thoughts and feelings about one’s own physical appearance [9], is generally regarded as one of the most robust predictors of a subsequent eating disorder [10, 11] and has also been linked with greater levels of suicidal ideation [12–14]. Notably, these relationships may be bidirectional; among eating disorder patients, suicidal ideation predicts subsequent eating disorder symptoms, even after accounting for current symptom severity [15]. Taken together, these findings suggest that eating pathology and suicidality may maintain and exacerbate one another via continuous feedback loops.

Individuals with ANSD and suicidality also share common comorbidities. Anxiety disorders are substantially more prevalent among individuals with eating disorders than the general population [16], and these disorders often predate the onset of AN [17]. Anxiety disorders are also associated with increased risk of suicidal thoughts and behaviors, with one study finding that the presence of an anxiety disorder at baseline yields a threefold increase in risk for a subsequent suicide attempt [18]. For individuals with ANSD, experiencing heightened anxiety in the context of limited emotion regulation skills may result in the adoption of maladaptive emotion regulation strategies [19], including disordered eating [20] and/or suicidal behaviors [21]. Therefore, anxiety and emotion regulation difficulties may serve to maintain both eating disorder symptoms and suicidality among individuals with ANSD by contributing to feedback loops which further entrench these conditions.

## Suicide within an Ideation-to-Action framework

Several suicide theories [22–24] propose that suicidal behaviors arise in the context of an ideation-to-action framework. Within this framework, the factors that precipitate suicidal ideation are distinct from those that precipitate suicidal behaviors; in other words, there are specific factors that distinguish ideators (i.e., those who experience suicidal ideation) from attempters (i.e., those who go on to engage in potentially lethal behaviors). Specifically, these theories posit that the capability for suicide, operationalized as a combination of fearlessness about death and elevated pain tolerance, is necessary for a suicide attempt to occur [24]. Suicidal capability is challenging to assess [25, 26], however, which makes it difficult to formally test these theories.

In addition to relatively stable traits such as the capability for suicide, transient physiological states may provide insight into the progression from suicidal ideation to attempts. Over-arousal, a bodily state characterized by agitation and irritability, has been implicated in the transition to suicidal behavior and may serve as an indicator of acute suicide risk [27]. Sleep disturbances, particularly insomnia, are also considered warning signs for imminent suicidal behavior [28]. These phenomena have been integrated into a clinical entity termed Acute Suicidal Affective Disturbance (ASAD; [29], which is a syndrome that involves over-arousal (e.g., insomnia, irritability) in the context of increasing suicidal intent, social or self-alienation, and hopelessness. ASAD is viewed as distinct from suicidal capability; however, among individuals who possess the capability for suicide, over-arousal may increase the likelihood of suicidal behavior by providing the energy needed to engage in lethal actions [30].

Limited research has explored the progression of suicidal ideation to suicide attempts in individuals with ANSD. This represents a major gap in research, as many of the factors associated with the transition from ideation to lethal behavior are commonly observed in ANSD, including suicidal capability [31], disturbed sleep [32], hyperactivity [33], and agitation [34]. Many of these symptoms may also arise as a consequence of body dissatisfaction, which has been independently linked to poor sleep [35] and the lethality and quantity of lifetime suicide attempts [36]. Other correlates of ANSD and suicidality, including anxiety and emotion dysregulation, have also been relatively under-explored in terms of their relationship with suicidal behaviors within ANSD. Notably, both anxiety and emotion dysregulation can lead to increased physiological arousal, suggesting that over-arousal may be one mechanism by which these symptoms increase risk for suicide in ANSD.

## Suicidality as a dynamic process

Despite decades of research, reliable short-term predictors of suicide remain elusive [37]. Most studies examine the predictive ability of putative risk factors over months or years [38], which makes it challenging to determine when to implement appropriate risk mitigation strategies in the short-term. These challenges are compounded by the fact that suicidality is a dynamic phenomenon, with fluctuations in ideation observable over the course of only a few hours [39]. Many putative predictors of suicide are also dynamic and state-based [40]. Therefore, methodological approaches capable of capturing volatility in suicidality and its risk factors are necessary to determine when risk for engagement in suicidal behaviors is highest, particularly in vulnerable populations such as those with ANSD.

Physiological data, including metrics such as Heart Rate Variability (HRV), Electrodermal Activity (EDA), and accelerometry, hold great promise for enhancing suicide prediction by capturing dynamic changes in risk processes as they occur. HRV, a measure of variability in the interval between consecutive heartbeats, is associated with the ability to effectively modulate emotional responses to stressful experiences [41]. HRV may therefore be a useful index of momentary changes in emotion regulation abilities. Notably, HRV has also demonstrated associations with suicidality [42, 43], negative body image [44], and maladaptive eating behaviors [45], further highlighting its potential utility as a risk marker for suicidality and disordered eating. Accelerometry, a method that allows for the objective assessment of physical activity, can be used evaluate sleep disturbances, which are known risk factors for suicide [46]. Indeed, emerging research suggests that actigraphic sleep parameters are predictive of changes in suicidal ideation over a period as brief as one week [47]. Because sleep disturbances are characteristic of over-arousal states associated with elevated suicide risk, actigraphic assessment of sleep may also clarify when a progression from suicidal ideation to suicidal behavior is likely to occur [48]. EDA, or variations in the skin’s electrical properties, is another useful index of over-arousal [49] which may enhance our understanding of high-arousal states that facilitate the transition from ideation to action [50] via passive sensing.

Although physiological data alone may be insufficient to accurately predict suicide, combining objective and subjective assessments of physiological and psychological states appears to enhance the prediction of suicidal thoughts and behaviors [51]. Cutting-edge data analytic techniques (e.g., network analysis, machine learning) have the potential to clarify how physiological and psychological processes work together to maintain eating pathology and confer risk for suicidality in ANSD.

## Individual symptom dynamics in ANSD and suicide

Eating disorders and suicidality are conditions characterized by substantial heterogeneity [52, 53] and complexity [54, 55]. Developing interventions that effectively address concomitant ANSD and suicidality necessitates understanding how various symptoms work together to maintain these conditions, while simultaneously accounting for individual differences in clinical presentation. Network theory, a novel approach to the conceptualization of mental disorders, offers one data-driven framework for understanding how symptoms and physiology interact as components of a complex system [56].

**Fig. 1:**
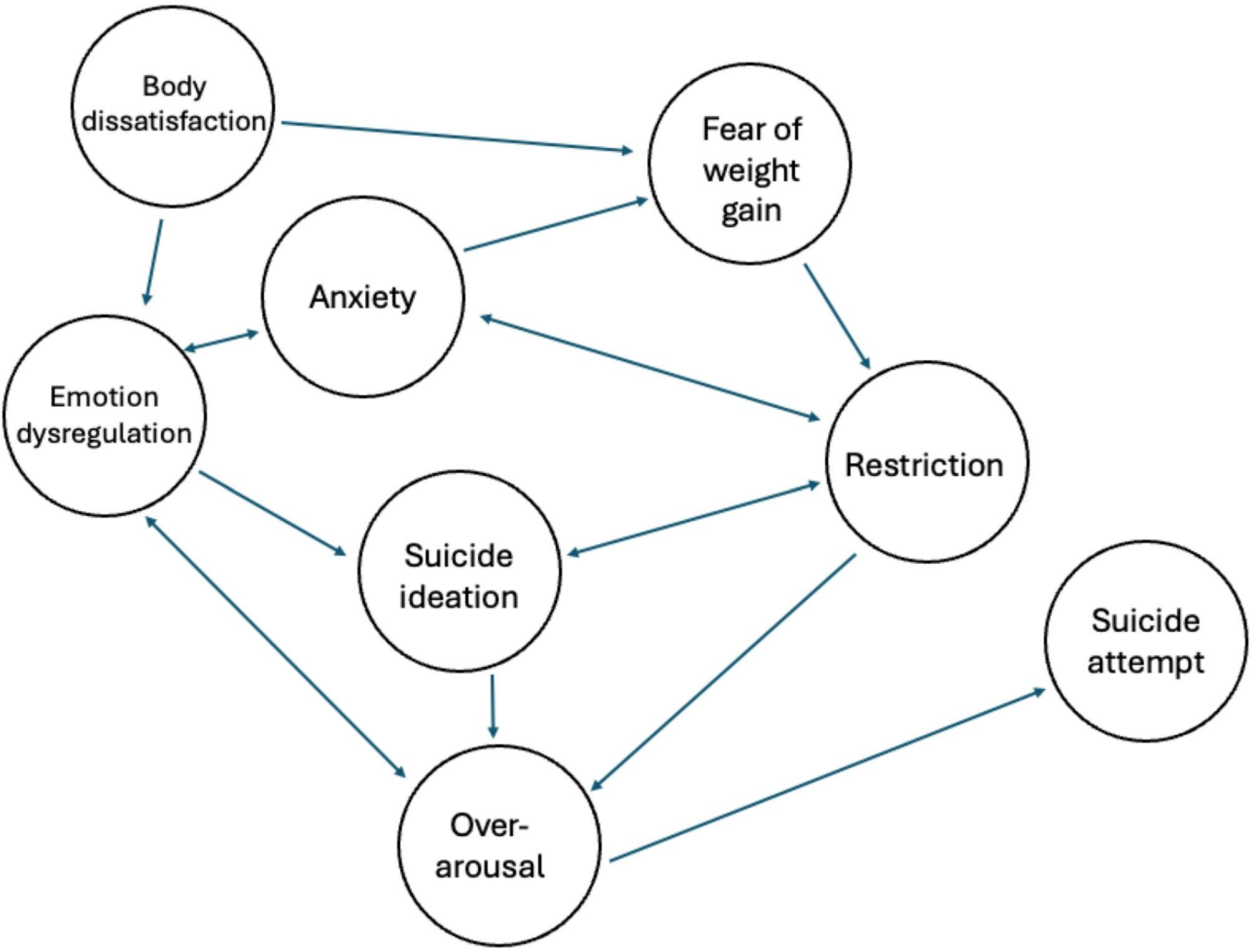
Conceptual Model of the Progression from Suicidal Ideation to Attempts in ANSD

**Fig. 2:**
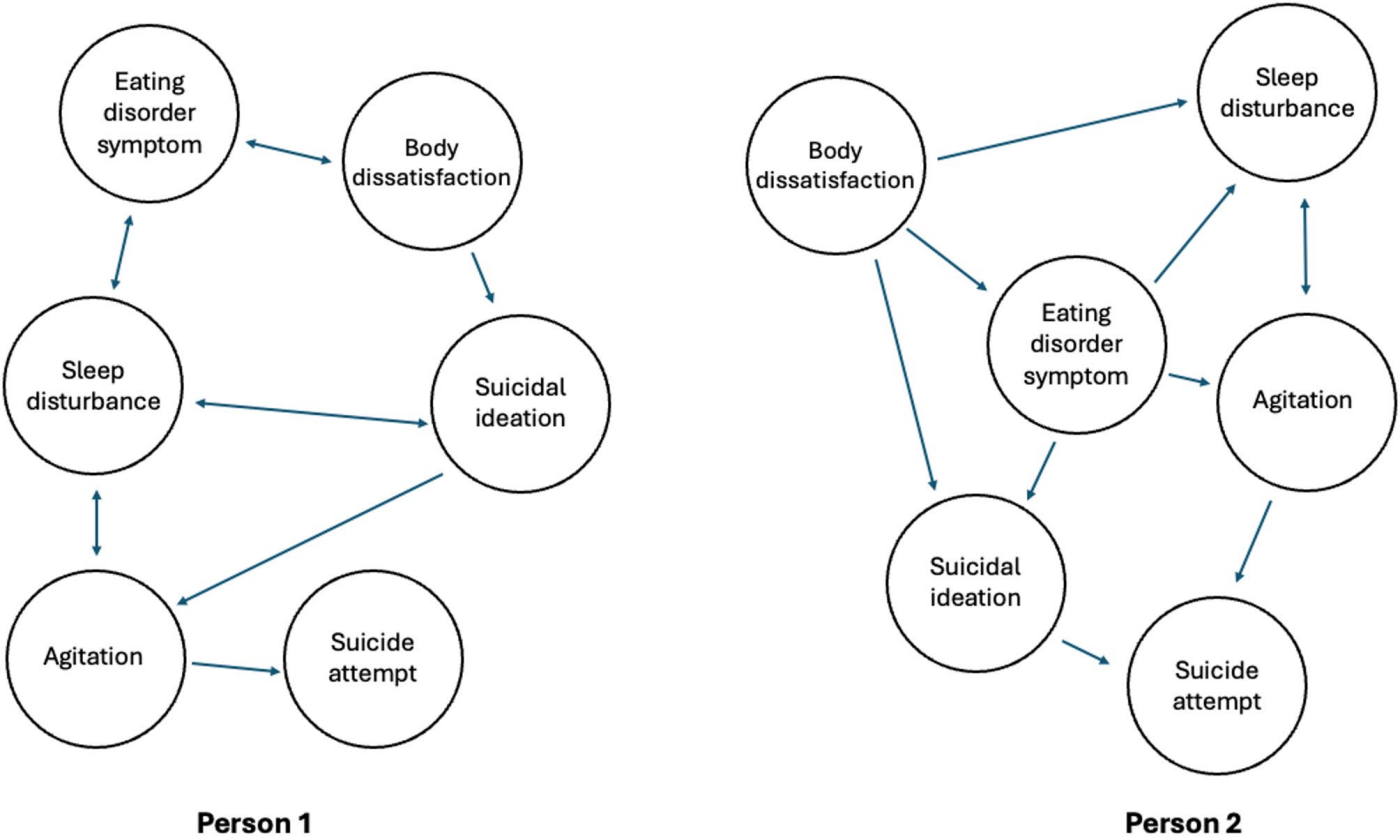
Differences in Relationships between Core Network Features across Persons

The statistical application of network theory is known as network analysis. Using symptom-level data, network analysis allows for the identification of “central nodes” (i.e., core features) which are highly connected to other symptoms, as well as “bridges” that link discrete syndromes to one another [57]. Central nodes are theorized to be the most viable intervention targets, as intervening on core disorder features should perturb the entire network of symptoms [58]. Core features are important predictors of treatment outcomes [59], suggesting that they may also be relevant to the prediction of adverse outcomes, including suicidal ideation and attempts. Critically, network analysis can be used to understand how symptoms interact at both the group and individual level [58], thereby making it possible to differentiate between shared and unique pathways to suicide in ANSD. Figure 1 depicts a conceptual model of the progression from suicidal ideation to suicide attempts in ANSD, and Fig. 2 demonstrates how the relationships between core features may vary from person-to-person. Ultimately, this approach has the potential to illuminate viable nomothetic and idiographic treatment targets, as well as to enhance the prediction and prevention of suicide in ANSD.

## The present study

The overarching aim of the present study is to identify the behavioral, physiological, and psychological mechanisms linking ANSD and suicide and to build models of suicide risk. Integrating data across multiple levels of analysis, we will develop group-level and personalized (i.e., idiographic) networks that will allow us to identify features that (a) maintain comorbid ANSD and suicidality, and (b) predict future suicidal ideation and suicide attempts among individuals with ANSD, both between and within persons.

Based on prior research and preliminary data, we hypothesize that body dissatisfaction, dietary restriction, and anxiety will be central features of ANSD that also predict suicidal ideation and attempts among individuals with these disorders. We expect that overarousal and suicidal ideation will maintain comorbid ANSD and suicidal behavior, and that these features will also predict subsequent suicide attempts. We anticipate that core symptoms will vary substantially across individuals, with systematic differences between those who do and do not engage in suicidal behavior, and we hypothesize that individual core features will predict both suicidal ideation and suicide attempts at follow-up. We further hypothesize that between-group models will not vary based on diagnosis (i.e., AN vs. AAN). Lastly, using a combination of passive sensing and EMA data, we expect that we will be able to build models that predict suicidal ideation and attempts with moderate-to-high accuracy.

## Methods

### Participants

We will recruit 230 adults with ANSD primarily from eating disorder specialty clinics, local and national eating disorder centers, and psychiatric and emergency hospitals. Participants will also be recruited via fliers, advertisements, social media posts, and recruitment websites (e.g., ResearchMatch, BuildClinical).

#### Inclusion criteria

Eligible participants must be between the ages of 18 and 65 and meet DSM-5 criteria [1] for AN or AAN. To meet criteria for AN, participants must be at less than 90% of their ideal body weight [60]; however, individuals with AN in partial remission (i.e., no longer underweight but still demonstrating clinically significant eating disorder symptoms) will also be eligible for inclusion. One-third of the sample will be required to have a history of suicide attempts, and two-thirds of the sample will be required to have either current or past suicidal ideation.

#### Exclusion criteria

Participants will be excluded if they demonstrate active psychosis, current mania, serious suicidal intent requiring immediate hospitalization, or if they are medically compromised and unable to safely complete study procedures.

### Procedure

**Fig. 3:**
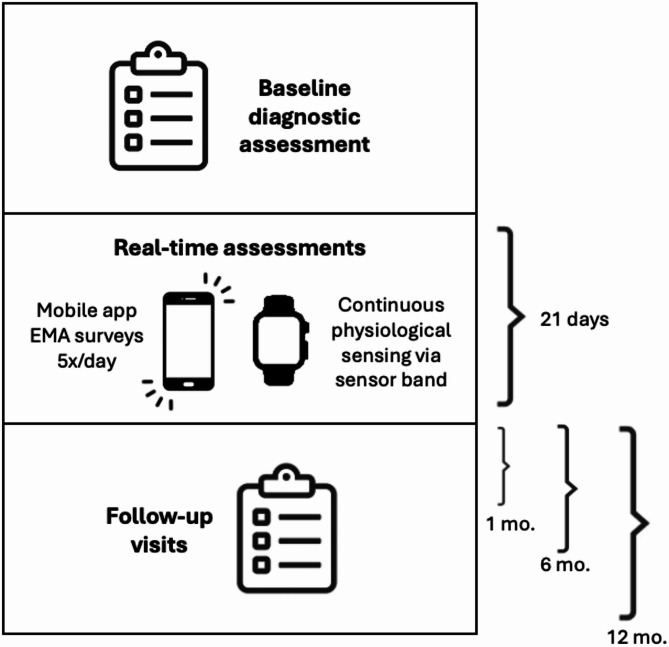
General Study Procedures

All procedures were approved by the University of Louisville Institutional Review Board (23.0356). General procedures are depicted in Fig. 3, and the schedule of assessments is reported in Table 1.

**Table 1:** Schedule of assessments

Assessment	Baseline	Week 1	Week 2	Week 3	1-month	6-month	12-month
MINI	x						
SCID-5	x				x	x	x
EDDI	x				x	x	x
SITBI-R	x				x	x	x
Weight	x				x	x	x
Demographics	x						
EDE-Q	x				x	x	x
EPSI	x				x	x	x
BDI-II	x				x	x	x
DERS	x				x	x	x
PROMIS Anxiety	x				x	x	x
ASI-3	x				x	x	x
ISI	x				x	x	x
PSQI	x				x	x	x
DDNSI	x				x	x	x
BAM	x				x	x	x
PANAS	x						
Sleep diary		x	x	x			
Wearable sensor		x	x	x			
EMA		x	x	x			

#### Baseline assessment

After completing a brief online eligibility screener, all participants will undergo a baseline structured clinical interview with a trained study coordinator to establish eligibility and assess current eating disorder symptoms, suicidality, current height and weight, comorbid psychiatric conditions, and treatment history. Following their baseline interview, participants will complete a battery of self-report measures assessing demographic characteristics, eating disorder symptoms, body image concerns, over-arousal symptoms, anxiety, emotion regulation difficulties, and other potentially relevant symptoms and traits (e.g., social appearance anxiety).

#### Suicide risk assessment

All participants, regardless of their level of suicide risk, will be provided with a personalized safety card at baseline that includes contact information for mental health professionals and relevant emergency resources (e.g., the Suicide Prevention Lifeline). Any participant with recent (i.e., past week) suicidal behavior or current suicidal intent will undergo an additional comprehensive suicide risk assessment, and actions will be taken to mitigate risk according to an empirically supported decision tree [61].

#### Ecological momentary assessment and passive sensing wearable band

Following baseline procedures, participants will complete 21 days of ecological momentary assessment (EMA). Surveys will be sent to participants’ phones five times per day, with each survey lasting approximately three minutes. During this 21-day period, participants will also be sent and instructed to wear an Empatica Embrace Plus sensor band, which will passively assess HRV, EDA, and accelerometry in real time. In addition to continuous passive sensing, participants will be instructed to create a “tag” consisting of one button press on the Empatica band any time they eat or exercise, and two button presses when they engage in disordered eating behaviors (e.g., binge eating, purging) or self-harm (e.g., self-cutting, engaging in suicidal behavior). Please see “Measures: Physiological Data” for additional information.

#### Follow-up assessments

Participants will complete follow-up assessments (including clinical interviews and self-report measures) at one-month, six months, and 12 months to assess any changes in eating disorder symptoms or suicidality since baseline.

#### Participant compensation

Participants will receive $20 for completing the baseline assessment and will receive up to an additional $200 for completion of the EMA and sensor band portion of the study. Specifically, for EMA surveys, participants will be compensated based on the number of surveys they complete, as follows: $100 for less than 50% of surveys, $120 for 50–60% of surveys, $140 for 61–70% of surveys, $160 for 71–80% of surveys, $180 for 81–90% of surveys, and $200 for 90% or more of surveys. Participants will receive an additional $50 for each follow-up assessment they complete, for a total of up to $350 per participant.

## Measures

### Clinical interviews

#### Mini International Neuropsychiatric Interview (MINI)

 The MINI [62] is a brief semi-structured diagnostic interview used to assess DSM-5 psychiatric disorders. It will be administered to assess for the possible presence of mania, psychosis, depressive disorders, Social Anxiety Disorder, Generalized Anxiety Disorder, Post-Traumatic Stress Disorder, Obsessive-Compulsive Disorder, Panic Disorder, Agoraphobia, and Specific Phobia. Participants with comorbid diagnoses will remain eligible for inclusion, with the exception of those with active mania or psychosis.

#### Structured Clinical Interview for DSM-5 (SCID-5) 

The SCID-5 [63] is a semi-structured diagnostic interview based on DSM-5 criteria for psychiatric disorders. The eating disorder modules of the SCID (i.e., Anorexia Nervosa, Bulimia Nervosa, Binge Eating Disorder) will be administered to establish eating disorder diagnoses for all participants.

#### Eating Disorder Diagnostic Interview (EDDI)

 The EDDI [64] is a semi-structured interview that assesses the presence and frequency of specific eating disorder symptoms, including binge eating, purging, laxative and diuretic use, exercise, and weight and shape concerns. The EDDI will be used to ensure the accuracy and specificity of eating disorder diagnoses as determined by the SCID-5 and to assess symptoms with additional granularity.

#### Self-Injurious Thoughts and Behaviors Interview – Revised (SITBI-R) 

The SITBI-R [65] is a semi-structured interview assessing the presence and characteristics of a range of self-injurious behaviors, including non-suicidal self-injury, suicidal ideation, suicide plans, and suicide attempts. The SITBI-R will be used to determine eligibility and to establish the presence of current and/or lifetime suicidal thoughts and behaviors.

### Self-report measures

#### Demographics and treatment history

 At baseline, participants will self-report basic demographic characteristics including age, sex assigned at birth, gender, race/ethnicity, and household income. We will also assess current use of psychotropic medication, treatment history, and illness duration. These variables will be used as covariates in our analyses.

#### Eating Disorder Examination Questionnaire (EDE-Q)

 The EDE-Q [66] is a 22-item self-report assessment of eating disorder symptoms. Responses to EDE-Q can be used to generate a global eating disorder severity score, as well as subscale scores for Restraint, Eating Concern, Weight Concern, and Shape Concern.

#### Eating Pathology Symptom Inventory (EPSI) 

The EPSI [67] is a 45-item self-report measure of eating pathology. Responses to the EPSI can be used to generate scores on eight subscales: Body Dissatisfaction, Binge Eating, Cognitive Restraint, Purging, Restricting, Excessive Exercise, Negative Attitudes toward Obesity, and Muscle Building.

#### Beck Depression Inventory-II (BDI-II)

 The BDI-II [68] is a 21-item self-report measure of the presence and severity of depressive symptoms. Depression severity will be included as a covariate in analyses to ensure that any suicide-related effects are independent of depressive symptomatology.

#### Difficulties in Emotion Regulation Scale (DERS)

 The DERS [69] is a 36-item multidimensional assessment of emotion regulation and dysregulation. It comprises six factors which relate to distinct components of emotion regulation difficulties, including Nonacceptance of Emotional Responses, Difficulties Engaging in Goal-Directed Behavior, Impulse Control Difficulties, Lack of Emotional Awareness, Limited Access to Emotion Regulation Strategies, and Lack of Emotional Clarity.

#### Patient-Reported Outcomes Measurement Information System (PROMIS) Anxiety Scale 

The PROMIS Anxiety Scale [70] is a self-report measure of anxiety-related symptoms, including fear, hyperarousal, anxious misery (e.g., dread), and somatic arousal symptoms (e.g., dizziness).

#### Anxiety Sensitivity Index-3 (ASI-3)

 The ASI-3 [71] is an 18-item self-report measure of anxiety sensitivity, or fear of sensations related to physiological arousal. It assesses arousal-related fears across three domains: physical concerns, cognitive concerns, and social concerns.

#### Insomnia Severity Index (ISI) 

The ISI [72] is a brief, seven-item screening measure used to detect the possible presence of insomnia. It assesses a variety of sleep-related issues, including nocturnal and early morning awakenings, sleep satisfaction, interference with daily functioning, and impairment and distress related to sleep problems.

#### Pittsburgh Sleep Quality Index (PSQI)

 The PSQI [73] is 19-item self-report assessment of sleep quality and disturbances. It can be used to generate seven component scores: sleep quality, sleep latency, sleep duration, sleep efficiency, sleep disturbances, sleep medication use, and daytime dysfunction.

#### Disturbing Dreams and Nightmares Severity Index (DDNSI)

 The DDNSI [74] is a self-report measure of the frequency and severity of nightmares and disturbing dreams. It comprises two factors: nightmare frequency and nightmare distress.

#### Brief Agitation Measure (BAM) 

The BAM [75] is a three-item measure of past-week agitation. Based on evidence suggesting that acute suicidal states are characterized by over-arousal, this measure was designed as a screener for imminent suicide risk.

#### Positive and Negative Affect Schedule (PANAS)

 The PANAS [76] is a combination of two 10-item scales assessing positive and negative affect, respectively. Unlike EMA items which primarily assess state-level affect, the PANAS measures both state (i.e., current) and trait (i.e., general) experiences of positive and negative affect.

### EMA

EMA surveys will assess eating disorder symptoms, suicidal thoughts and behaviors, affect, sleep-related symptoms, and over-arousal. Questions will ask about current levels of symptoms or engagement in behaviors since the previous assessment. All EMA items will be rated on a continuous 0–100 scale to ensure adequate variance for the estimation of network models. Please see Table 2 for a complete list of constructs assessed and example items for each construct.

**Table 2:** List of constructs assessed via EMA

Construct	# of items	Content	Example item
Eating disorder symptoms	19	Urge to skip meals, urge to restrict, restriction, food rules, food avoidance, skipping meals, vomiting, laxatives, binge eating, excessive exercise	*“I have tried to follow definite rules regarding eating in order to influence my shape or weight”*
Suicidal ideation	10	Passive ideation, active ideation, method, controllability of thoughts, suicide as solution to problems, desire to kill self	*“I have been thinking about killing myself”*
Suicidal behavior	3	Urges to self-harm, self-harm behaviors, suicidal behaviors	*“Have you done anything to try to kill yourself?”*
Anxiety	2	Worry, nervousness	*“I feel worried”*
Sleep	16	Time to sleep, restlessness, sleep quality, difficulty falling asleep, difficulty staying asleep, early awakening, sleep satisfaction, disturbing dreams	*“How much difficulty did you have falling asleep?”*
Agitation	3	Crawling out of skin, feeling stirred up, emotional turmoil	*“I feel so stirred up inside I want to scream”*
Affect	10	Alertness, determination, attentiveness, fear, hostility, shame	*“I feel determined”*

### Physiological data

All physiological data will be collected using Empatica Embrace Plus sensor bands [77], which were originally developed to improve seizure detection via ambulatory monitoring. Empatica bands have also been used in prior research to detect the onset of disordered eating behaviors [78]. Using the Empatica sensor bands, we will continuously record blood volume pulse, EDA, and accelerometry. All physiological sensor data will be time-aligned with self-report symptom measures to prepare the data for entry into machine learning algorithms.

#### HRV

 Empatica bands use photoplethysmography, a non-invasive optical method for tracking blood flow dynamics through light absorption changes, to yield information regarding HRV. We will use Kubios software [79] and custom Python scripts [80] to derive heart rate and HRV indices from blood volume pulse data.

#### EDA

 EDA reflects fluctuations in the skin’s electrical characteristics, primarily driven by changes in conductance due to sweat gland activation, and serves as an indicator of sympathetic nervous system activity. Empatica software utilizes a proprietary algorithm to distinguish meaningful autonomic events (i.e., tonic EDA), from autonomic changes attributable to environmental factors (e.g., temperature, movement).

#### Accelerometry

 Because of the correlation between sleep stages and motor activity, accelerometers – which measure changes in physical activity – can be used to objectively assess a variety of sleep metrics. Accelerometry data will be passively collected via Empatica bands, and the three vector indices will be combined via Fourier transformation into a single consolidated vector. A validated sleep scoring algorithm [81] will be used to determine total sleep time, sleep onset latency, frequency and duration of awakenings after sleep onset, and sleep efficiency (i.e., the percentage of time spent asleep while in bed).

### Physical metrics

Participants will self-report their current height, weight, and weight history during the baseline assessment. To maximize reliability, participants will be asked to use the same scale at each assessment point. For the small number of participants who may not have access to a scale, a scale will be provided via mail. Weight history and current weight will be used to calculate weight suppression [82], or the difference between the participant’s current and highest weight. These data will be used as a proxy for medical and nutritional status, as well as to evaluate the potential impact of weight loss and/or gain on eating disorder symptoms and suicidality.

## Data analytic plan

### Maintenance of ANSD and suicidality

#### Network Estimation Procedure

**s** Multilevel vector autoregressive network models will be estimated using the *mlVAR* package [83] in R [84]. These models leverage intensive longitudinal data to provide insight into symptom dynamics at both the group (i.e., between-person) and individual (i.e., within-person) level. In addition to differentiating between within- and between-person processes, network models can be used to understand the relationships between symptoms (or “nodes”) at the same timepoint (i.e., contemporaneously) or longitudinally, and to identify which symptoms are most important to the structure of the entire network (i.e., central symptoms). We will estimate a series of different network models to address our specific research aims.

For all networks, we will begin by including eight symptoms (e.g., eating disorder symptoms, agitation, anxiety), as well physiological data (i.e., HRV, EDA, and accelerometry). The eight included symptoms for nomothetic networks will be the symptoms with the highest means across the entire sample; for idiographic networks, they will be the symptoms with the highest means for each person. Model fit for each network will be evaluated using the *psychonetrics* package [85]. If model fit is determined to be poor, the number of symptoms included in each model will be adjusted using the *Goldbricker* function from the *networkTools* package [86], which can be used to identify potentially redundant nodes.

#### Identification of Core Features 

To test which symptoms and core disorder features maintain comorbid ANSD and suicidality at the group and individual level, we will estimate nomothetic and idiographic temporal (i.e., longitudinal) networks, respectively. These procedures will generate a unique contemporaneous and temporal idiographic network for each participant in the study, as well as nomothetic contemporaneous and temporal networks representing the entire sample. For all networks, we will identify core features based on “out-strength,” a centrality index which quantifies the influence of a particular symptom on other symptoms in the network [57]. The symptoms with the highest centrality (i.e., largest out-strength values) will be used as independent variables in the prediction of suicide-related outcomes, as described below. Network group invariance testing will be used to compare whether network structure systematically differs as a function of diagnosis (i.e., AN vs. AAN) across idiographic temporal models [87].

### Prediction of suicidal ideation and attempts

#### Group-level Prediction 

The symptoms and features with the highest centrality in nomothetic networks will be evaluated as potential predictors of suicidal ideation and suicide attempts at follow-up. Specifically, we will employ multivariate logistic regression to determine the extent to which these central features predict suicidal ideation and suicide attempts at one-month, six-month, and 12-month follow-up.

#### Individual-level Prediction

 To test whether idiographic network features enhance the prediction of suicide-related outcomes at the individual (*N* = 1) level, we will leverage a cutting-edge machine learning approach. Specifically, we will employ personalized deep learning models known as “long short-term memory networks,” or LTSM, which are a type of recurrent neural network [88] to predict the likelihood of suicidal ideation and suicide attempts based on individual-level demographic data (e.g., age, body mass index), symptom-level data (e.g., baseline EDE-Q scores), and central network features.

### Power analysis and missing data

#### Network Estimation 

Given the relative novelty of network analytic approaches, there are no universally accepted power analysis guidelines for determining an appropriate sample size [89]. However, power for network models can be understood as a function of sample size, number of timepoints, and the number of features included in the model. Given our expected sample size (*N* = 230), number of timepoints (i.e., 105), and number of nodes (i.e., 8), prior research by our group suggests that we should have excellent power to detect significant relationships between symptom nodes in group-level analyses [90].

For idiographic models, because the sample size is always *N* = 1, we are primarily concerned with stability, rather than power. Stability can be thought of as a form of reliability [91]. In prior work [92], we have achieved excellent stability generating idiographic networks with up to 15 included features across 105 timepoints. Because this study will also generate 105 timepoints per participant, and we anticipate including fewer than 15 features in each model, we expect that our idiographic models to demonstrate comparable, if not greater, stability.

Missing data are common in EMA studies, and we anticipate a missing data rate of approximately 25%. If networks do not converge due to missing data or inadequate variability, we will employ a Kalman filter using the package *imputeTS* [93] for imputation.

#### Regression Analyses

 We used G*Power [94] to determine power for our regression analyses predicting suicidal ideation and suicide attempts. Suicide attempts are fortunately much rarer than suicidal ideation, even in high-risk populations (e.g., individuals with a history of attempts). However, due to our stratified sampling strategy, which prioritizes the recruitment of participants with either current or past suicidality, we anticipate a suicide attempt incidence rate of approximately 15–25% over the course of the study. Regression analyses will include three central symptoms as independent variables, as well as 3–5 demographic covariates. We expect to recruit 230 participants, which would ensure power of at least 0.80 to detect a small effect size (i.e., 0.20) even with a drop-out rate of approximately 25% (i.e., final *N* = 172).

## Discussion

The proposed research combines intensive longitudinal data collection, passive sensing of physiological symptoms, and novel advances in network science to identify the behavioral, physiological, and psychological mechanisms linking ANSD and suicide. Specifically, we aim to elucidate dynamic longitudinal interactions among core disorder features (e.g., body dissatisfaction, over-arousal) that contribute to the development of suicidal ideation and suicide attempts at both the group and individual level. Findings from this project will facilitate the development of highly personalized intervention strategies that effectively prevent suicidal behaviors. For example, this study may lead to the creation of wearable sensors capable of alerting users and their healthcare providers to instances of increased suicide risk, streamlining the delivery of just-in-time interventions and emergency services that prevent suicidal behaviors before they occur. Findings from this study will also allow us to develop effective, novel, personalized treatments that address core symptoms of ANSD in real time.

## Data Availability

Data will be available via the National Institute of Mental Health Data Archive.

## Abbreviations

AAN: Atypical anorexia nervosa
AN: Anorexia nervosa
ANSD: Anorexia nervosa spectrum disorder
ASAD: Acute suicidal affective disturbance
ASI: Anxiety Sensitivity Index
BAM: Brief Agitation Measure
BDI: Beck Depression Inventory
DDNSI: Disturbing Dreams and Nightmares Severity Index
DERS: Difficulties in Emotion Regulation Scale
EDA: Electrodermal activity
EDDI: Eating Disorder Diagnostic Interview
EDE-Q: Eating Disorder Examination Questionnaire
EMA: Ecological momentary assessment
EPSI: Eating Pathology Symptom Inventory
HRV: Heart rate variability
ISI: Insomnia Severity Index
MINI: Mini International Neuropsychiatric Interview
PROMIS: Patient-Reported Outcomes Measurement Information System
PANAS: Positive and Negative Affect Schedule
PSQI: Pittsburgh Sleep Quality Index
SCID: Structured Clinical Interview for DSM
SITBI: Self-Injurious Thoughts and Behaviors Interview
